# ^18^F-FLT PET/MRI for bone marrow failure syndrome-initial experience

**DOI:** 10.1186/s13550-019-0490-0

**Published:** 2019-02-15

**Authors:** Tetsuya Tsujikawa, Toshiki Tasaki, Naoko Hosono, Tetsuya Mori, Akira Makino, Yasushi Kiyono, Paolo Zanotti-Fregonara, Takahiro Yamauchi, Hidehiko Okazawa

**Affiliations:** 1 Biomedical Imaging Research Center, University of Fukui, 23-3 Matsuoka-Shimoaizuki, Eiheiji-cho, Fukui, 910-1193 Japan; 2 Department of Hematology and Oncology, Faculty of Medical Sciences, University of Fukui, 23-3 Matsuoka-Shimoaizuki, Eiheiji-cho, Fukui, 910-1193 Japan; 3 Houston Methodist Research Institute, Weill Cornell Medicine, 6670 Bertner Ave, Houston, TX 77030 USA

**Keywords:** Bone marrow failure syndrome, PET/MRI, ^18^F-FLT, DWI

## Abstract

**Background:**

Bone marrow failure syndrome (BMFS) is a heterogeneous group of disorders associated with single- or multiple-lineage cytopenia and failure of normal hematopoiesis. We assessed the feasibility of integrated PET/MRI with 3′-deoxy-3′-^18^F-fluorothymidine (^18^F-FLT) to assess the pathophysiology of whole-body bone marrow for the diagnosis and monitoring of BMFS. Twenty-five consecutive patients with BMFS underwent a pre-treatment ^18^F-FLT PET/MRI scan. They included 7 patients with aplastic anemia (AA), 16 with myelodysplastic syndrome (MDS), and 2 with myeloproliferative neoplasms (MPNs), primary myelofibrosis (MF), and secondary [post-essential thrombocythemia (post-ET)] MF. Two of the seven AA patients underwent a post-treatment scan. Eight of the 16 MDS patients who exhibited decreased ^18^F-FLT uptake in the pelvis were considered to have hypoplastic MDS (hypo-MDS). ^18^F-FLT PET and diffusion-weighted imaging (DWI) were visually and quantitatively evaluated.

**Results:**

The ^18^F-FLT uptake in the ilium was strongly correlated with bone marrow cellularity based on biopsy samples (ρ = 0.85). AA patients exhibited heterogeneously decreased uptake of ^18^F-FLT according to disease severity. Multiple ^18^F-FLT foci were observed in the proximal extremities, and they were in the central skeleton in severe AA patients. Post-treatment ^18^F-FLT PET scans of severe AA patients reflected the response of hematopoietic activity to treatment. MDS patients had marked ^18^F-FLT uptake in the central skeleton and proximal extremities, whereas hypo-MDS patients had heterogeneously decreased uptake, similar to that of non-severe AA patients. ^18^F-FLT PET and DWI were unable to predict the progression to leukemia for both MDS and hypo-MDS patients. A primary MF patient had slightly decreased ^18^F-FLT uptake in the central skeleton, but marked expansion of bone marrow activity to the distal extremities and high uptake of tracer in the extremely enlarged spleen (extramedullary hematopoiesis). In contrast, a secondary (post-ET) MF patient demonstrated marked bone marrow uptake, reflecting the hypercellular marrow with fibrosis. DWI revealed diffusely high signal intensities in both the primary and secondary MF patients.

**Conclusion:**

^18^F-FLT PET can be used to noninvasively assess whole-body bone marrow proliferative activity and DWI may reflect the different aspects of bone marrow pathophysiology from ^18^F-FLT PET. ^18^F-FLT PET/MRI is useful for the diagnosis and monitoring of BMFS, except for the differentiation between non-severe AA and hypo-MDS, and the prediction of progression to leukemia.

## Background

**Fig. 1:**
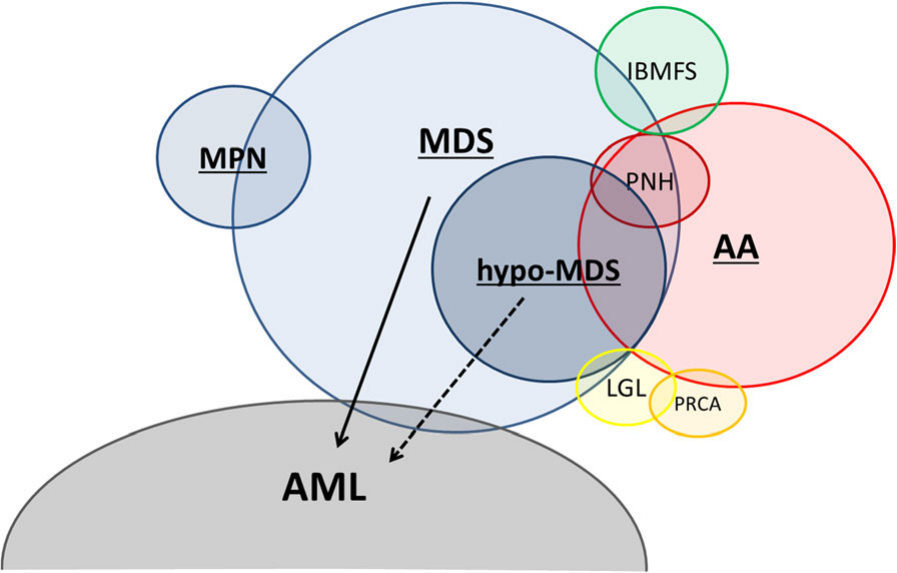
Overlap of bone marrow failure syndrome and progression to leukemia [1]. *AA* aplastic anemia, *MDS* myelodysplastic syndromes, *IBMFS* inherited bone marrow failure syndrome, *LGL* large granular lymphocytosis, *PRCA* pure red cell aplasia, *PNH* paroxysmal nocturnal hemoglobinuria, *MPN* myeloproliferative neoplasm, *AML* acute myeloid leukemia

Bone marrow failure syndrome (BMFS) is a heterogeneous group of disorders associated with single- or multiple-lineage cytopenia and failure of normal hematopoiesis that can be either inherited or acquired [1]. BMFS includes aplastic anemia (AA), myelodysplastic syndrome (MDS), inherited BMFS (IBMFS), large granular lymphocytosis (LGL), pure red cell aplasia (PRCA), paroxysmal nocturnal hemoglobinuria (PNH), and myeloproliferative neoplasms (MPNs) such as primary myelofibrosis (primary MF) (Fig. 1). There is some degree of overlap among these syndromes as two disorders can coexist in the same patient, and some of these disorders can evolve to acute myeloid leukemia (AML). Differential diagnosis can be difficult because these syndromes are primarily defined by the cell morphology based on limited bone marrow biopsy samples obtained from limited areas. However, misdiagnosis leads to inappropriate therapy and delayed treatment. Moreover, therapeutic monitoring can also be difficult because the evaluation is based on the limited biopsy samples, which do not reflect the response of whole-body bone marrow.

The thymidine analog 3′-deoxy-3′-^18^F-fluorothymidine (^18^F-FLT) is a radiopharmaceutical for positron emission tomography (PET) and a surrogate marker of DNA synthesis. ^18^F-FLT PET enables the evaluation of whole-body bone marrow proliferative activity, and has been used for the diagnosis and monitoring of bone marrow disorders [2–6]. Diffusion-weighted imaging (DWI) is a quantitative functional magnetic resonance imaging (MRI) technique that detects the random movement of water protons. Whole-body DWI is currently used for cancer staging and assessment of treatment response in malignancies involving bone marrow [7–10]. The recent introduction of integrated PET/MRI has enabled simultaneous PET and MR functional imaging with matched spatial registration and high soft tissue contrast [11].

In this study, we assessed the feasibility of whole-body ^18^F-FLT PET/MRI as a two-way imaging system comprising PET and DWI for the differential diagnosis and therapeutic monitoring of BMFS.

## Methods

### Healthy subjects

Before starting ^18^F-FLT PET/MR scans for patients, three healthy subjects underwent ^18^F-FLT PET/MRI to set the parameters for PET and MR sequences and to confirm the normal distribution of ^18^F-FLT and DWI signals.

### Patient population

Twenty-five patients (19 males and 6 females; 68.5 ± 16.3 years of age) with biopsy-confirmed BMFS, comprising 7 with AA, 16 with MDS, and 2 with MPNs consisting of primary MF and secondary (post-essential thrombocythemia; post-ET) MF, were enrolled in this feasibility study (Table 1). Bone marrow biopsy samples were obtained from the unilateral posterior iliac crest. AA patients were classified as having severe AA or non-severe AA according to the modified Camitta’s criteria [12, 13]. All patients underwent a pretreatment ^18^F-FLT PET/MRI scan, and two patients with severe AA underwent a post-treatment scan to assess the treatment response of bone marrow compartments between August 2016 and June 2018. This retrospective study was approved by the ethics committee of the Faculty of Medical Sciences, University of Fukui (No. 20160030). Written informed consent was obtained from all individual participants including healthy subjects in this study.

**Table 1:** BMFS patient characteristics

Patient	Age	Sex	Diagnosis	WBC	Hb	Plt	Bone marrow	FLT-SUV			DWI	Progress
				(× 10^3^/μL)	(g/dL)	(×10^3^/μL)	biopsy	Ilium	Lumbar	Sternum	Score	
1	88	M	Non-severe AA	5	7.1	113	Hypocellular	3.7	8.9	6.5	1	
2	63	F	Non-severe AA	2.4	12	64	Hypocellular	1.6	7.8	8.2	1	
3	81	M	Non-severe AA	1.9	5.1	117	Hypocellular	0.4	4.9	1.2	1	
4	24	F	Non-severe AA	2	11	12	Hypocellular	0.2	14.2	13.7	1	
5	79	M	Non-severe AA	2.7	10.3	35	Hypocellular	1	7.8	14.7	2	
6	66	F	Severe AA	1	5.2	25	Hypocellular	0.9	4.1	3.9	1	
7	67	M	Severe AA	3.1	5.6	4	Hypocellular	0.3	0.5	3.2	1	
8	70	M	MDS	1.3	5.8	34	Normocellular	7.9	11.4	13.9	1	
9	66	M	MDS	1.6	8.4	47	Normocellular	7.8	12.1	10.1	1	
10	80	M	MDS	3	6.8	105	Hypercellular	8.6	11.5	9.2	2	AML
11	68	F	MDS	7	6	13	Hypercellular	9.4	9.5	9.7	1	AML
12	68	M	MDS	1	7.2	26	Normocellular	5.1	5.3	7.1	1	AML
13	66	M	MDS	8.6	11.1	112	Normocellular	5	6.4	6.5	2	
14	65	F	MDS	2.5	7.1	37	Hypercellular	11.5	13	16.5	1	
15	81	F	MDS	6.3	10	74	Hypercellular	7.4	9.9	9.8	3	
16	18	M	hypo-MDS	0.8	7.5	121	Hypocellular	2.9	9.7	8.3	1	
17	77	M	hypo-MDS	1.7	8.3	20	Hypocellular	2.1	7.8	1.3	1	AML
18	68	M	hypo-MDS	3	10	133	Hypocellular	1.2	3	6.2	2	
19	72	M	hypo-MDS	1.5	9.9	5	Hypocellular	1	3.3	4.6	2	
20	60	M	hypo-MDS	1.3	8.3	154	Hypocellular	3.1	3.5	4.7	1	AML
21	75	M	hypo-MDS	2.5	12	36	Hypocellular	1.6	2.8	8.8	1	
22	81	M	hypo-MDS	2.5	8.3	39	Hypocellular	1.7	5.6	3.5	2	AML
23	90	M	hypo-MDS	2.7	7.9	319	Normocellular	3.1	6.1	7.5	2	
24	69	M	MPN (primary MF)	7	11.6	14	Normocellular	2.7	4.7	4	3	
25	70	M	MPN (secondary MF)	19.5	11.2	438	Hypercellular	9.5	16	15.1	3	

*M* male, *F* female, *AA* aplastic anemia, *MPN* myeloproliferative neoplasms, *MF* myelofibrosis, *MDS* myelodysplastic syndrome, *AML* acute myeloid leukemia, *WBC* white blood cell, *Hb* hemoglobin, and *Plt* platelet count

### Whole-body PET/MRI

#### ^18^F-FLT synthesis

^18^F-FLT was synthesized in a TRACERlab MXFDG (GE Healthcare) using an ABX-FLT kit (ABX) [14]. No-carrier-added ^18^F-fluoride was produced via the ^18^O(p,n)^18^F reaction from > 98% enriched ^18^O-water (Cambridge Isotope Laboratories) on an RDS eclipse RD/HP medical cyclotron (Siemens/CTI). The radiochemical purity of the final product was > 99%, and the yield was 12.3 ± 4.6% (end of synthesis, *n* = 17).

#### PET scan and Dixon-based MR-AC

Patients fasted for at least 4 h prior to an intravenous injection of 200 MBq of ^18^F-FLT. Fifty minutes after the injection, patients were transferred to the whole-body simultaneous 3.0 T PET/MR scanner (Signa PET/MR, GE Healthcare, Waukesha, WI, USA). Anatomic coverage was from the vertex to the mid-thigh. PET acquisition was performed in 3D mode with 5.5 min/bed position (89 slices/bed) in 5–6 beds with a 24-slice overlap. The 5.5 min/bed rate was selected to accommodate the MRI sequences acquired at each bed. A 2-point Dixon 3D volumetric interpolated T1-weighted fast spoiled gradient echo sequence (TR/TE1/TE2: 4.0/1.1/2.2 ms; FOV 50 × 37.5 cm; matrix 256 × 128; slice thickness/overlap: 5.2/2.6 mm; 120 images/slab; imaging time: 18 s) was acquired at each table position and used to generate MR attenuation correction (MR-AC) maps. The PET data were reconstructed using ordered subset expectation maximization (OSEM) selecting 14 subsets and 3 iterations, and post-smoothed with a 3-mm Gaussian filter. Reconstructed images were then converted to a semi-quantitative image corrected by the injection dose and subject’s body weight (= standardized uptake value: SUV).

#### DWI sequence parameters

DWI was performed using a single shot echoplanar imaging (EPI) sequence under free breathing (TR/TE: 5000/61 ms; *b* values: 0, 800 s/mm^2^; FOV 576 × 345 mm; matrix 128 × 128; slice thickness/overlap: 6/0 mm; 40 images/bed; imaging time: 2 min 30 s).

#### Visual and quantitative image assessment

Maximum intensity projection (MIP) images of ^18^F-FLT PET and DWI with *b* = 800 were used for the visual assessment of bone marrow. Eight of the 16 MDS patients who exhibited decreased ^18^F-FLT uptake in pelvic bones were considered to have hypoplastic MDS (hypo-MDS). ^18^F-FLT PET and DWI were visually compared among AA, MDS, hypo-MDS, and MPN (primary and secondary MF) patients.

For ^18^F-FLT PET quantitative assessment, circular regions of interest (ROIs) with a fixed diameter of 15 mm were placed on the bilateral posterior iliac crest and lumbar vertebrae (L3–5). Elliptical ROIs were placed on the manubrium and body of the sternum. ^18^F-FLT-SUVs were measured and averaged in the ilium, L3–5, and sternum by the agreement of an experienced radiologist and hematologist. For DWI, three-point visual scoring was used to evaluate whole-body bone marrow signals. A score of 1 was assigned to images in which bone marrow was invisible, a score of 2 to images in which bone marrow was partially visible, and a score of 3 to images in which bone marrow was fully visible. Regression analysis between bone marrow cellularity based on biopsy samples and ^18^F-FLT uptake in the ilium was performed using Spearman’s rank correlation coefficient (ρ). The SUVs and DWI visual scores were compared among diseases by analysis of variance with the post-hoc Games-Howell test and Kruskal-Wallis test, respectively. Statistical analysis was performed using a software package (SPSS statistics version 22) and *p* < 0.05 was considered to be significant.

## Results

### Normal ^18^F-FLT PET and DWI

**Fig. 2:**
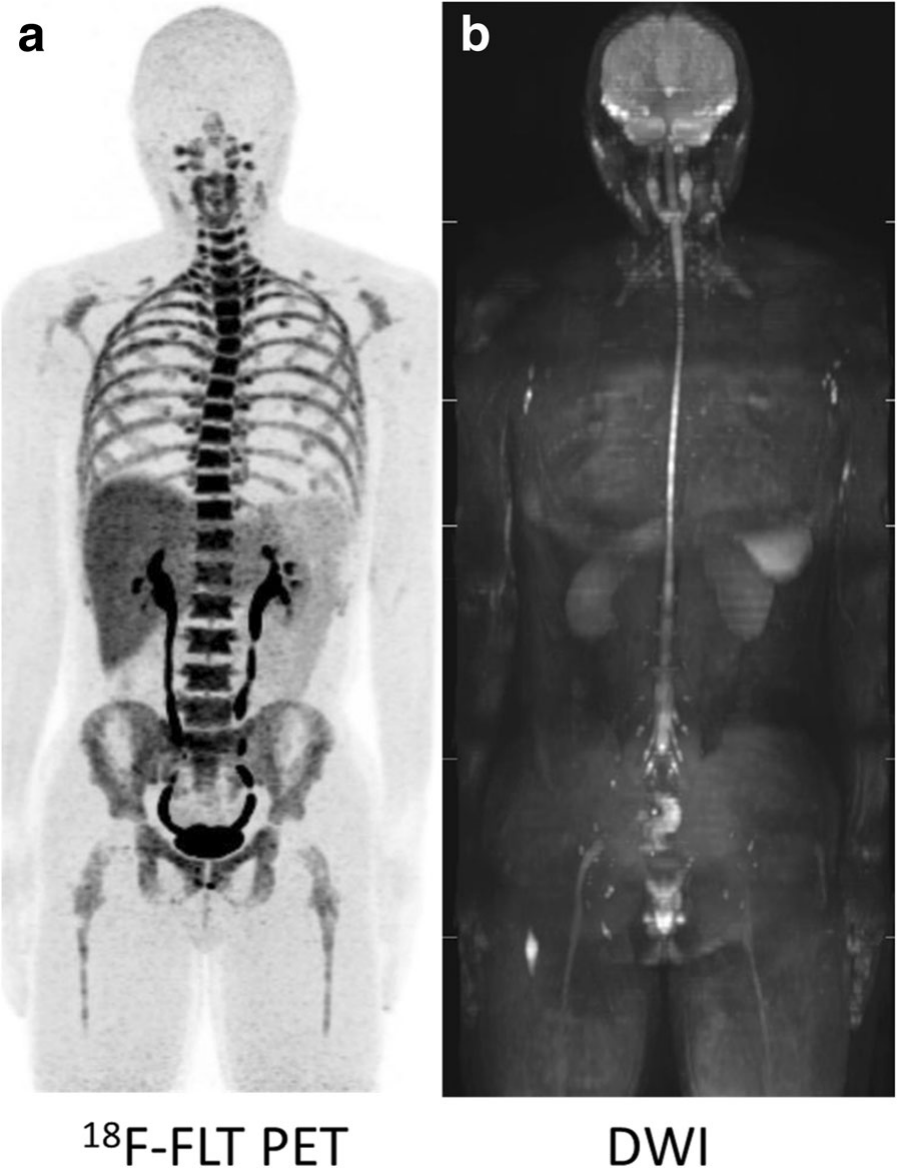
Maximum intensity projection ^18^F-FLT PET (**a**) and DWI (**b**) images of a healthy subject. ^18^F-FLT PET clearly visualizes whole-body hematopoietic activity consistent with red bone marrow distribution (**a**). Whole-body DWI visualizes some organs, such as the spleen, kidneys, spinal cord, and lymph nodes, whereas red bone marrow is invisible or partially visible in normal subjects (**b**)

^18^F-FLT PET clearly visualized whole-body bone marrow activity consistent with red bone marrow distribution (Fig. 2a). Whole-body DWI visualized some organs, such as the spleen, kidneys, spinal cord, and lymph nodes (Fig. 2b), whereas red bone marrow was invisible or partially visible in healthy subjects.

#### Bone marrow cellularity and ^18^F-FLT uptake

Bone marrow cellularity based on biopsy samples was classified into three categories: hypocellular, normocellular, and hypercellular (Table 1). All patients with AA (*n* = 7) exhibited hypocellular marrow, four of eight MDS patients were hypercellular, and the others had normocellular marrow. Seven of eight patients with hypo-MDS were hypocellular, and one patient had normocellular marrow. The primary MF patient was normocellular and the secondary MF patient had markedly hypercellular marrow. The ^18^F-FLT SUV of the ilium was strongly correlated with the bone marrow cellularity (ρ = 0.85).

#### Aplastic anemia

**Fig. 3:**
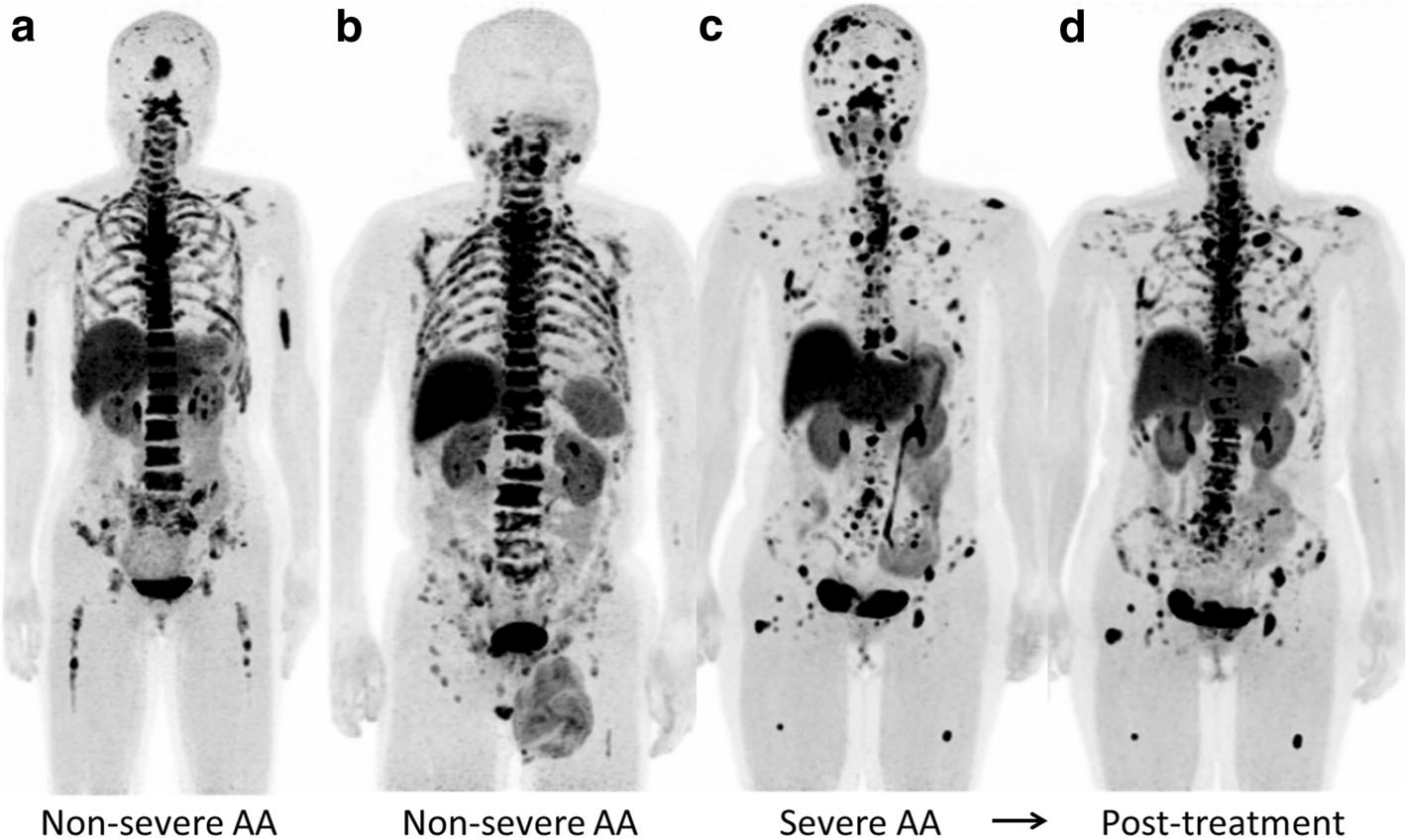
Maximum intensity projection ^18^F-FLT PET images of AA patients (**a**–**d**). Non-severe AA patients exhibited heterogeneously decreased ^18^F-FLT uptake in pelvic bones (**a**, **b**), and this reduced uptake extended to the vertebrae and ribs with multiple ^18^F-FLT foci in a severe patient (**c**). Whole-body ^18^F-FLT PET clearly visualized the recovery of bone marrow hematopoietic activity in the severe AA patient after treatment (**d**)

**Fig. 4:**
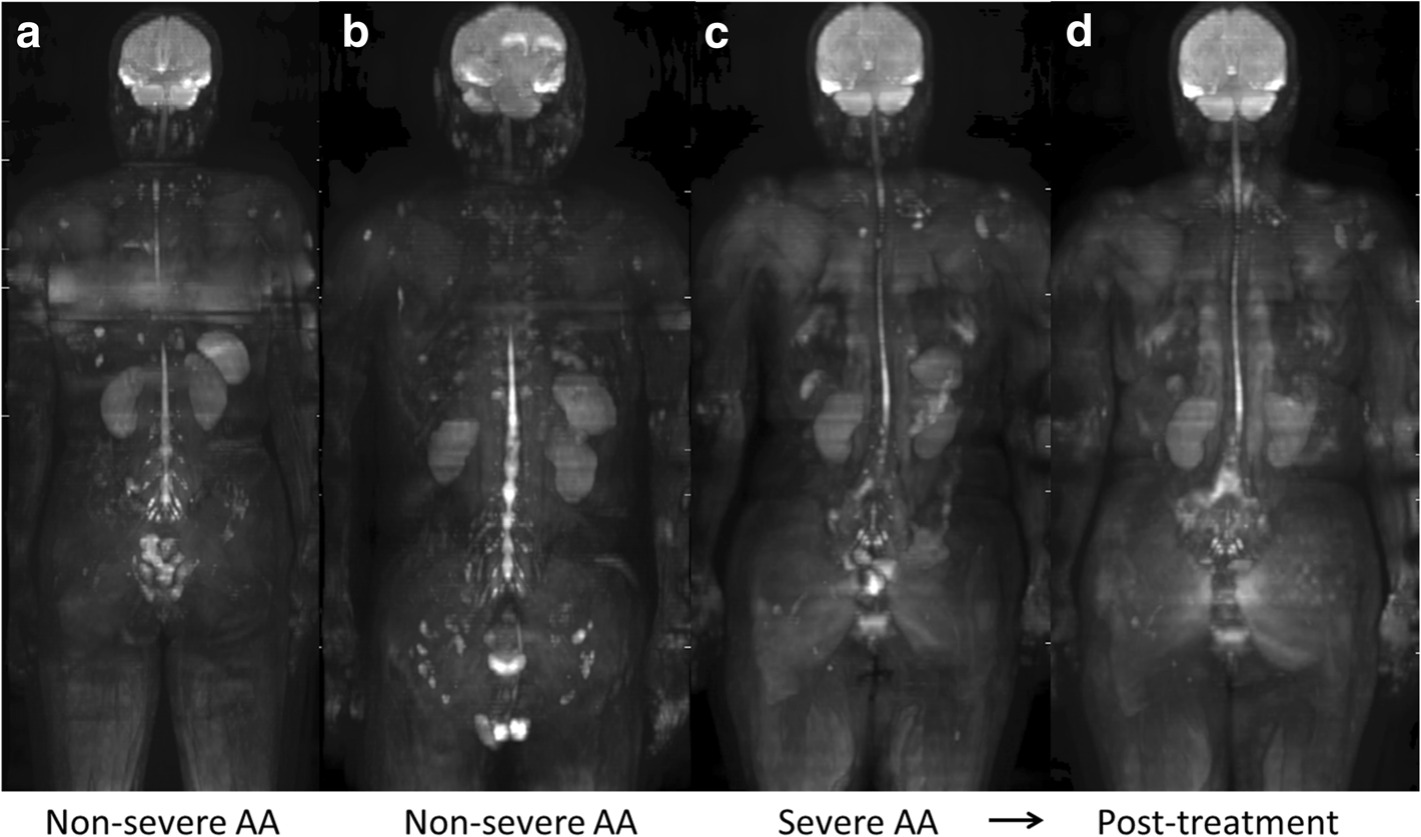
Maximum intensity projection DWI images of AA patients (**a**–**d**). **a**–**d** in Fig. 4 are identical to **a**–**d** in Fig. 3, respectively. Whole-body DWI did not demonstrate any significant differences in bone marrow signals between non-severe AA and severe AA patients, or between pre- and post-treatment scans

Non-severe AA patients exhibited heterogeneously decreased ^18^F-FLT uptake in pelvic bones (Fig. 3a, b), and this reduced uptake extended to the vertebrae and ribs in a severe patient (Fig. 3c). Multiple ^18^F-FLT foci were observed in the proximal extremities, and they were in the central skeleton in severe cases. In a patient with severe AA, post-treatment ^18^F-FLT PET demonstrated the recovery of hematopoietic activity in vertebrae (Fig. 3d), consistent with recovery of the blood count on blood testing. On the other hand, whole-body DWI did not provide any specific bone marrow findings for AA patients (Fig. 4).

#### Myelodysplastic syndrome

**Fig. 5:**
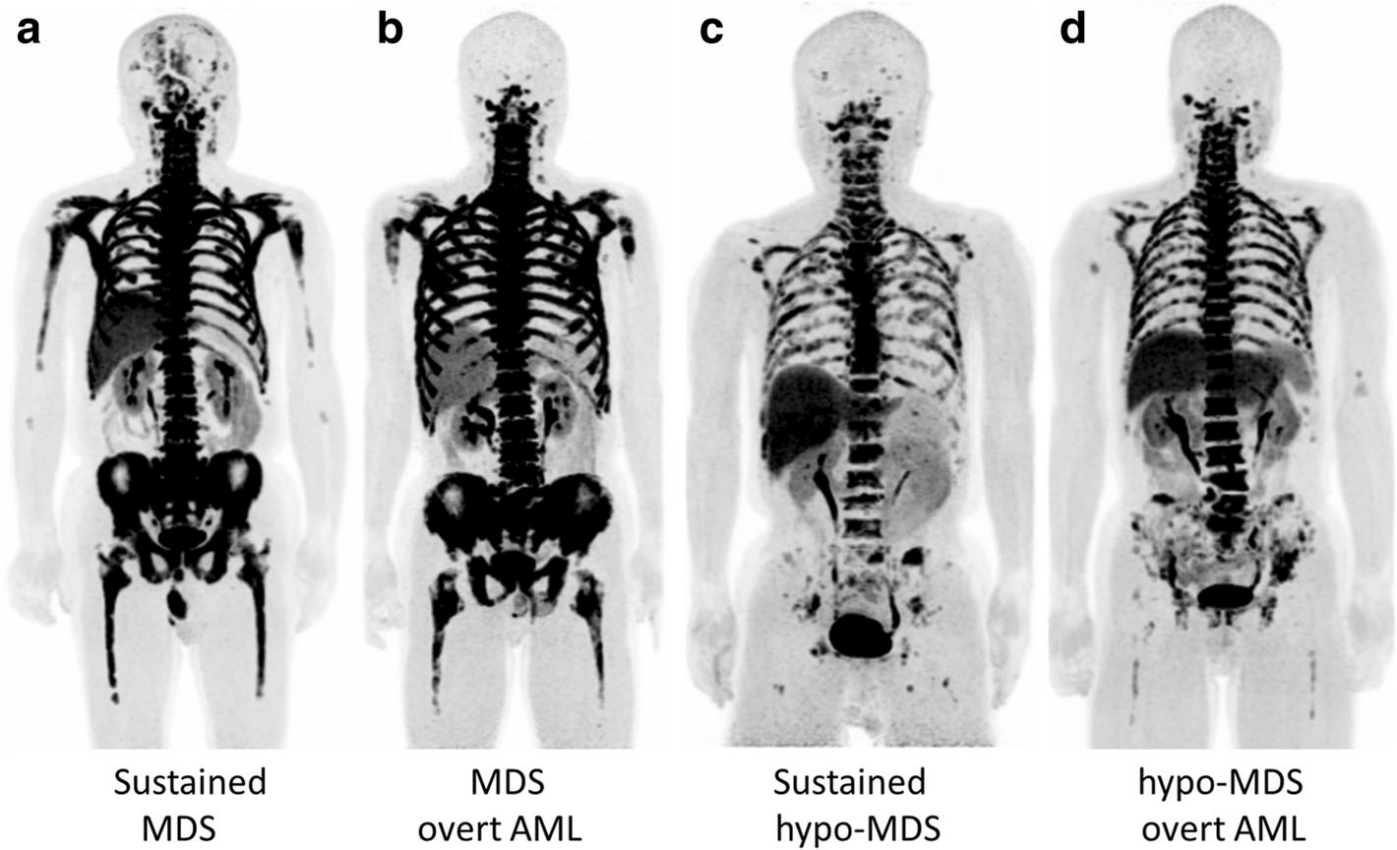
Maximum intensity projection ^18^F-FLT PET images of MDS patients (**a**–**d**). MDS patients exhibited marked ^18^F-FLT uptake in the central skeleton and proximal extremities (**a**, **b**). On the other hand, hypo-MDS patients who were defined by having decreased ^18^F-FLT uptake in the pelvis exhibited a slightly decreased uptake of the tracer in the vertebrae and ribs (**c**, **d**). ^18^F-FLT PET did not reveal any significant differences between patients with sustained MDS or overt AML

**Fig. 6:**
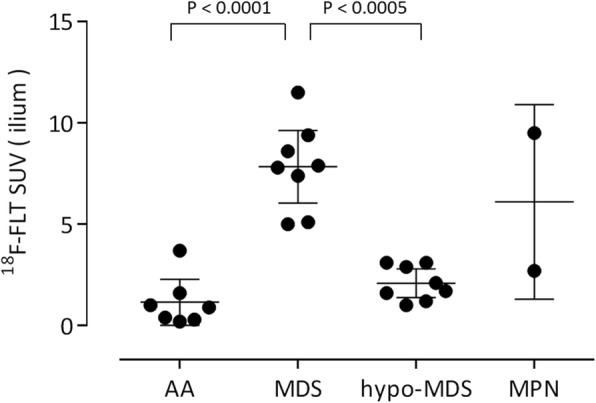
^18^F-FLT uptake in the ilium among AA, MDS, hypo-MDS, and MPN patients. Data are shown as the mean and 95% confidence interval for AA, MDS, and hypo-MDS patients, and as the mean and standard deviation for MPN patients. Significant differences in ^18^F-FLT uptake were observed between MDS and AA (*P* < 0.0001) or hypo-MDS patients (*P* < 0.0005)

**Fig. 7:**
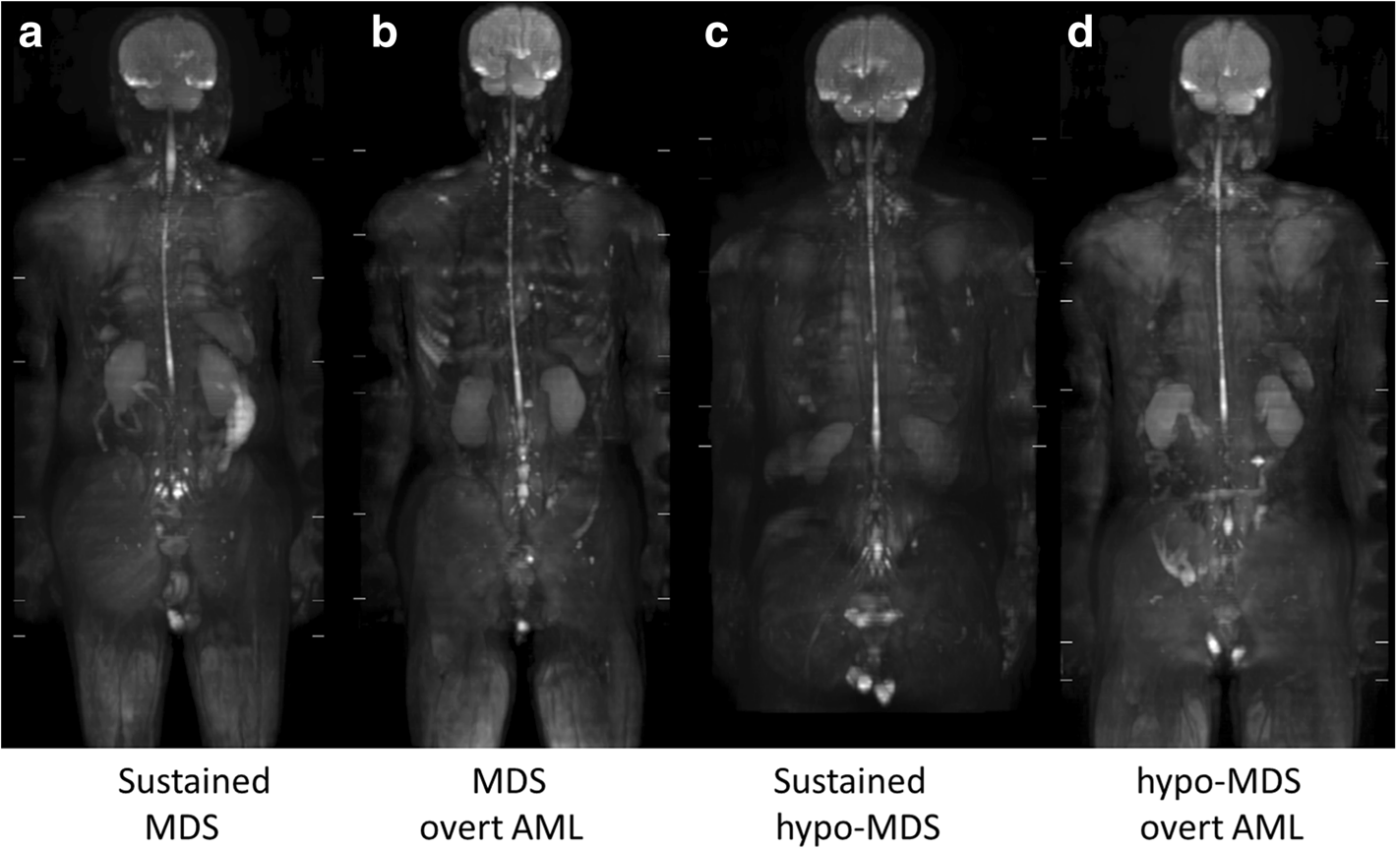
Maximum intensity projection DWI images of MDS patients (**a**–**d**). **a**–**d** in Fig. 7 are identical to **a**–**d** in Fig. 5, respectively. Whole-body DWI did not demonstrate any significant differences in bone marrow signals between MDS and hypo-MDS patients. DWI did not demonstrate any significant differences between patients with sustained MDS or overt AML

MDS patients exhibited marked ^18^F-FLT uptake in the central skeleton and proximal extremities (Fig. 5a, b). On the other hand, patients with hypo-MDS had slightly–moderately decreased ^18^F-FLT uptake in the vertebrae, ribs, and pelvis (Fig. 5c). ^18^F-FLT uptake in the central skeleton and proximal extremities was significantly higher in MDS patients than in hypo-MDS patients. The ^18^F-FLT PET findings in hypo-MDS patients were similar to those in non-severe AA patients (Figs. 3a, b and 5c, d). MDS patients had a significantly higher ^18^F-FLT SUV of the ilium than AA and hypo-MDS patients (*p* < 0.0001 and *p* < 0.0005, respectively) (Fig. 6). Whole-body DWI did not reveal any significant differences in bone marrow signals between MDS and hypo-MDS patients (Fig. 7). During the course of a disease, three of eight MDS patients (38%) and three of eight hypo-MDS patients (38%) developed AML. However, neither ^18^F-FLT PET nor DWI demonstrated any significant differences between patients with sustained MDS/hypo-MDS or overt AML (Figs. 5 and 7).

#### Myelofibrosis

**Fig. 8:**
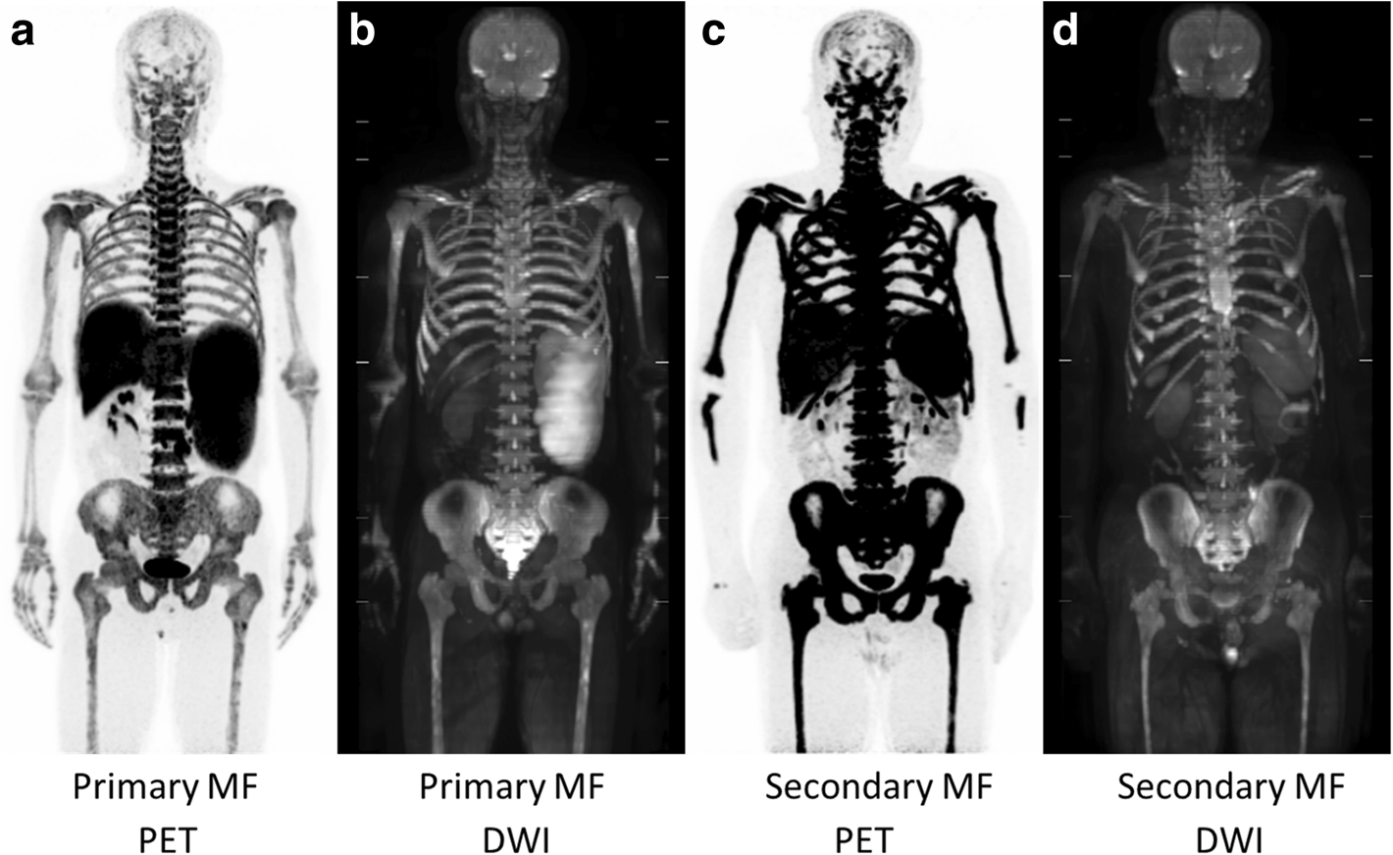
Maximum intensity projection ^18^F-FLT PET (**a**, **c**) and DWI (**b**, **d**) images of MF patients. A primary MF patient exhibited normal or slightly decreased bone marrow activity in the central skeleton, but they had marked expansion to the distal extremities and high uptake of the tracer in the enlarged spleen (**a**). On the other hand, a secondary (post-ET) MF patient had marked ^18^F-FLT uptake in bone marrow (**c**). Whole-body DWI displayed homogenously high signal intensities in bone marrow of primary and secondary MF patients (**b**, **d**, respectively)

In a primary MF patient, slightly decreased ^18^F-FLT uptake was observed in the central skeleton, possibly due to mild fibrosis confirmed by biopsy, but he had marked expansion of bone marrow activity to the distal extremities (Fig. 8a). In addition, high uptake of the tracer was observed in the enlarged spleen due to extramedullary hematopoiesis. In contrast to the primary MF patient, a secondary (post-ET) MF patient exhibited marked ^18^F-FLT uptake in the central skeleton and extremities (Fig. 8c). The biopsy samples from this post-ET MF patient exhibited significant fibrosis and markedly hypercellular bone marrow. High uptake of the tracer was also observed in the enlarged spleen in the secondary MF patient. Homogenously high signal intensities in bone marrow and splenomegaly in primary and secondary MF patients were noted on whole-body DWI (Fig. 8b, d). By group comparison, a significant difference in the DWI score was observed between AA and MF patients (*p* < 0.05).

## Discussion

In this feasibility study, we demonstrated the usefulness of ^18^F-FLT PET/MRI for the differential diagnosis and therapeutic monitoring of BMFS. ^18^F-FLT uptake in the ilium was strongly correlated with the bone marrow cellularity based on the biopsy samples in this patient population. AA patients exhibited heterogeneously decreased uptake of ^18^F-FLT according to disease severity and the response of hematopoietic activity to treatment (Fig. 3) [3]. In contrast to AA patients, MDS patients had homogeneous marked ^18^F-FLT uptake reflecting neoplastic proliferation (Fig. 5a, b) [2]. MF patients had marked expansion of bone marrow activity to the distal extremities and high uptake of the tracer in the enlarged spleen, reflecting peripheral hematopoietic expansion and extramedullary hematopoiesis, respectively (Fig. 8) [4]. The primary MF patient exhibited slightly decreased bone marrow activity, whereas the secondary MF patient had marked ^18^F-FLT uptake in bone marrow. The difference in bone marrow ^18^F-FLT uptake between the primary and secondary (post-ET) MF patients was probably due to the different histopathologies of the bone marrow. Although bone marrow biopsy demonstrated different degrees of fibrosis in the primary and secondary MF patients, markedly hypercellular marrow including megakaryocytes was observed in the secondary (post-ET) MF patient. Whole-body ^18^F-FLT PET can be used for non-invasive assessment of bone marrow hematopoietic/neoplastic activity and therapeutic monitoring for patients with bone marrow disorders [3–6]. Furthermore, if possible, whole-body imaging including the lower limbs is preferable for the accurate evaluation of peripheral bone marrow expansion.

The signal intensities of bone marrow on DWI are dependent on the cell density, the relative content of fat and marrow cells, water content, and bone marrow perfusion [15, 16]. Although whole-body bone marrow DWI has been used for assessing treatment response in patients with multiple myeloma [10, 17], to the best of our knowledge, no previous investigation has focused on bone marrow DWI in patients with BMFS. The MF patients exhibited characteristic diffuse high signal intensity on DWI (Fig. 8b, d), and had significantly higher DWI scores than AA patients. However, the other patients (AA, MDS, and hypo-MDS) did not exhibit any specific bone marrow signals on DWI. Among seven patients with AA, six patients had a DWI visual score of 1 (invisible) and one patient had a score of 2 (partially visible) (Table 1, Fig. 4). Although the low signal intensity of bone marrow on DWI can be explained by fatty degeneration, further studies are needed to identify the reason why the DWI signal intensity is low even in the areas of multiple ^18^F-FLT foci. Although ^18^F-FLT uptake patterns were significantly different between MDS and hypo-MDS patients, further analysis is needed to identify why their DWI signals were similar (Table 1, Figs. 5, 6, and 7). Differences in effects of cell/fat density, water content, and bone marrow perfusion should be clarified among AA (fatty bone marrow), MDS (neoplastic proliferation), and primary/secondary MF (fibrosis) in future studies. Whole-body DWI may reflect the different aspects of the bone marrow pathophysiology from ^18^F-FLT PET in patients with BMFS.

To our knowledge, no previous report of ^18^F-FLT PET for MDS has included hypoplastic MDS (hypo-MDS) [2], which presents reduced ^18^F-FLT uptake in pelvic bones, vertebrae, and ribs. Nakao et al. recently noted the similarity between mild/moderate AA and low-risk MDS, and that it is impossible to definitively distinguish these conditions [18, 19]. However, accurate differentiation is required because patients diagnosed with mild/moderate AA often receive immunosuppressive drugs, whereas those diagnosed with low-risk MDS often receive hypo-methylating drugs. Although hypo-MDS defined by reduced ^18^F-FLT uptake in pelvic bones is not strictly identical to low-risk MDS, hypo-MDS demonstrated similar ^18^F-FLT PET uptake patterns with non-severe AA in this study. Although the multiple ^18^F-FLT foci in the proximal extremities in AA patients may aid in the differentiation between non-severe AA and hypo-MDS, differentiation is sometimes difficult (see Figs. 3b and 5c, d). Moreover, ^18^F-FLT PET and DWI were unable to predict the progression to AML in both MDS and hypo-MDS patients (Figs. 5 and 7). Integrated PET/MRI that can extract mineable PET and MR radiomic features, and subsequent radiomics approaches may enable the differentiation of mild/moderate AAs from hypo-MDS, as well as the prediction of progression to AML in the near future [20].

## Conclusions

^18^F-FLT PET can be used to noninvasively assess whole-body bone marrow proliferative activity and DWI may reflect the different aspects of bone marrow pathophysiology from ^18^F-FLT PET. Whole-body ^18^F-FLT PET/MRI is useful for the diagnosis and monitoring of BMFS, except for the differentiation between non-severe AA and hypo-MDS, and the prediction of progression to leukemia. Further studies will be required to establish non-invasive pathophysiological imaging of whole-body bone marrow by integrated ^18^F-FLT PET/MRI.

## Abbreviations

^18^F-FLT: 3′-Deoxy-3′-^18^F-fluorothymidine
AA: Aplastic anemia
BMFS: Bone marrow failure syndrome
DWI: Diffusion-weighted imaging
EPI: Echoplanar imaging
ET: Essential thrombocythemia
hypo-MDS: Hypoplastic myelodysplastic syndrome
LGL: Large granular lymphocytosis
MDS: Myelodysplastic syndrome
MF: Myelofibrosis
MIP: Maximum intensity projection
MPNs: Myeloproliferative neoplasms
MR-AC: MR attenuation correction
MRI: Magnetic resonance imaging
OSEM: Ordered subset expectation maximization
PET: Positron emission tomography
PNH: Paroxysmal nocturnal hemoglobinuria
PRCA: Pure red cell aplasia
SUV: Standardized uptake value
